# Universal prevention for non-suicidal self-injury in adolescents is scarce - A systematic review

**DOI:** 10.3389/fpsyt.2023.1130610

**Published:** 2023-10-23

**Authors:** Arne Bürger, Cornelia von Schoenfeld, Christin Scheiner, Alexandra Seidel, Antonia Wasserscheid, Doreya Gad, Sarah Kittel-Schneider, Marcel Romanos, Andrea M. F. Reiter

**Affiliations:** ^1^Department of Child and Adolescent Psychiatry, Psychosomatics, and Psychotherapy, Center of Mental Health, University Hospital of Würzburg, Würzburg, Germany; ^2^German Centre of Prevention Research in Mental Health, University of Würzburg, Würzburg, Germany; ^3^Department of Psychiatry, Psychosomatics and Psychotherapy, Center of Mental Health, University Hospital of Würzburg, Würzburg, Germany; ^4^Acute Adult Mental Health Unit, Department of Psychiatry and Neurobehavioural Science, Cork University Hospital, Cork, Ireland; ^5^Department of Psychology, University of Würzburg, Würzburg, Germany

**Keywords:** non-suicidal self-injury, NSSI, emotion regulation, prevention, universal prevention, adolescence, mental health

## Abstract

Non-suicidal self-injury (NSSI) during adolescence is a high-risk marker for the development and persistence of mental health problems and has been recognized as a significant public health problem. Whereas targeted prevention has indeed shown to be effective in reducing NSSI and improve mental health problems, access to such programs is limited. By face validity, universal prevention of NSSI seems an ideal starting point for a stepped-care model to circumvent a lack of resources in the medical care system. However, it is yet unclear how effective such approaches are. Here, we provide a summary of existing work on universal prevention of NSSI in adolescents younger than 21 years based on a systematic literature search. We found that only seven studies are available. None of the programs evaluated was found to be effective in reducing the incidence or frequency of NSSI. After providing a comprehensive summary of the existing work, we evaluate the fact that existing work primarily focusses on selected/targeted prevention and on psychoeducational methods. We derive implications for future directions in the field of universal prevention of NSSI.

## Introduction

Non-suicidal self-injury (NSSI) is characterized by the intentional and self-inflicted destruction of body tissue without suicidal intent (1). According to the WHO (2), NSSI represents the fifth most frequent health risk in adolescence, with a lifetime prevalence estimated to be 18% for at least one self-injuring event in community samples worldwide (3). Brunner et al. (4) showed in a comparative study in 11 European countries that 7.8% of adolescents suffered from repetitive NSSI (≥ 5 acts during lifetime). NSSI shows increasing prevalence at age 13–14, peaking at around age 15–16 (5). NSSI may thus be triggered by puberty and the confrontation with multiple developmental challenges, including a substantial biological and social reconfiguration happening in this period of life which has been argued to trigger mood instability (6, 7). Above all, repetitive NSSI is a high-risk marker and a predictor of suicidal thoughts and behaviors, comorbid psychopathology (e.g., Depression, anxiety disorders, posttraumatic stress disorder, borderline personality disorder) and other high-risk behaviors (8–11). Furthermore, NSSI leads to high rates of hospitalization, resulting in high costs to the healthcare system (12). Consequently, NSSI was proposed as a new diagnostic entity in the fifth edition of the Diagnostic and Statistical Manual (DSM 5) in Section 3 (conditions for further investigation) (13). Thus, given the high prevalence of NSSI, its clinical significance and the substantial costs for the healthcare system it causes (12, 14), there is an urgent need for action to prevent it. Early adolescence represents a particularly suitable period for early prevention before first manifestations of NSSI occur, in order to protect against NSSI and the associated risk for mental health issues and suicidality (15).

Encouragingly, targeted prevention has been shown not only to significantly reduce NSSI, but also to improve mental health outcomes in adolescents with NSSI and suicidality (16, 17). However, access to these approaches is limited due to a lack of resources (e.g., effective interventions are only regionally available) and an insufficient number of specially trained clinicians (18). A possible alternative would be to prevent the development of NSSI, prior to the age of 11 to 14 years through universal prevention programs before adolescents begin to show these dangerous behaviors. NSSI is frequently reported significantly earlier than suicidal ideations and attempts (19). Thus, there is an urgent need to strengthen the focus on NSSI during early adolescence in line with the objective of universal prevention.

Universal approaches delivered to a broad population of youth are particularly beneficial in improving mental well-being and quality of life, and have been shown to be effective in reducing the incidence and costs of a range of chronic mental illnesses (20, 21). Yet, in general, research on universal prevention is sparse compared to the field of targeted prevention or therapeutic interventions. To the best of our knowledge, there is no systematic overview covering universal prevention approaches and their effectiveness in preventing NSSI. We therefore aimed to conduct a review including universal prevention programs in NSSI to determine: (i) which universal prevention programs exist, and (ii) how effective they are in reducing NSSI.

## Materials and methods

Following PRISMA guidelines (22), we performed a systematic literature research. We systematically searched the databases PubMed (1960–March 23th 2023) and Google Scholar (1960–March 23th 2023) for English or German language articles on universal prevention programs of NSSI with the following search terms: (“non-suicidal self-injury” OR “self-injury” OR “self-harm” OR NSSI OR Suicide) AND (prevent*) AND (adolescent* OR child* OR youth). For details on the search string, see Supplementary material. We chose the databases (PubMed and Google Scholar) in accordance with Kothgassner et al. (17), who conducted a systematic review and meta-analysis on therapeutic interventions for self-harm in adolescents in 2021. Even if the focus of this review was deliberately set on prevention of NSSI, the decision to include the search term “suicide” and “self-harm” was taken in order to allow for a broad search and to initially cover a broad range of studies which sometimes do not clearly differentiate NSSI from suicidal thoughts and behaviors. We also aimed to include older studies, which historically used these terms even when implying non-suicidal intentions. Subsequently, we screened all references in the publications obtained from step 1 for further relevant articles. After removing duplicates, we screened titles and abstracts. If studies were relevant to the topic, we obtained the full texts. To fulfill the objective of this review, i.e., to focus on NSSI without suicidal motives, studies were screened and included into the analysis only when providing data on NSSI independent of suicidality. In the next step, studies were reviewed in sections using the PICO model, thus evaluating studies for participants, interventions, comparisons and outcomes.

### Study selection

Study designs were allowed for inclusion regardless of whether they were controlled or uncontrolled, as long as they met the criteria of either randomized controlled trials, pre-post or cohort-studies. Studies were eligible if they focused on universal prevention of NSSI and targeted a general population sample of adolescents outside a clinical context. Regarding the age cut off, samples addressing adolescents with a median age under 21 years were included. In order to fulfill our inclusion criteria, interventions needed to be designed and carried out with a specific focus on the prevention of NSSI (allowing heterogeneous kinds of preventive approaches), i.e., without suicidal intent. Furthermore, for a study to be included, it was required that it was made clear to study participants that the prevention program was targeting NSSI (as distinct from suicidality). Further, outcome measures needed to focus on NSSI actions (occurrence, frequency, method) or knowledge (attitudes, handling, coping skills). Again, it had to be clearly evident to participants that the measurement was about self-injury without suicidal intent. If the focus on NSSI was not clear either in the program itself or in the measurement instruments used, the studies were excluded. No specific data collection method was determined necessary for inclusion, as guidelines on a standardized specific survey of NSSI are currently lacking.

### Data extraction

Included studies were independently screened by at least two authors (AB, AW, ChS, CoS, DG) and entered into a spreadsheet (1st step: title + abstract, 2nd step: full text). Disagreement was resolved through discussion.

### Data synthesis

**FIGURE 1:**
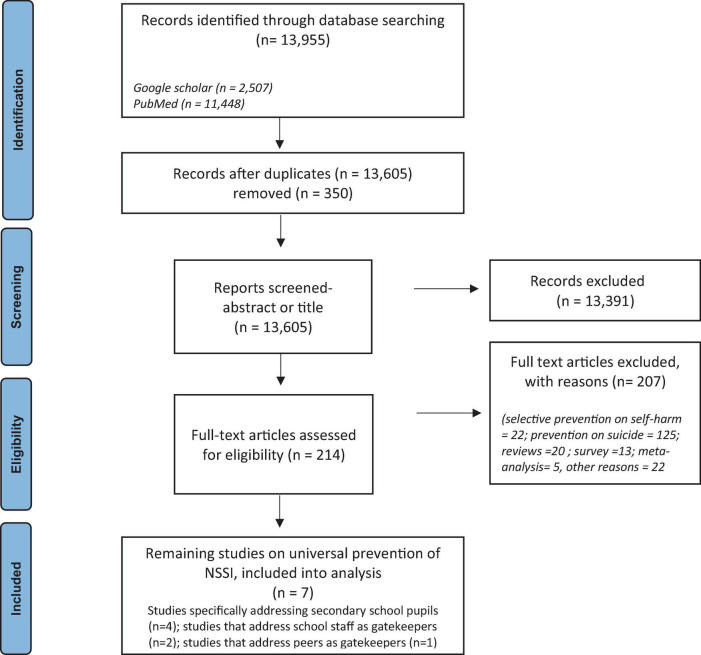
Flow chart according to PRISMA guidelines (46).

We identified 13,955 studies after removal of duplicates (*k* = 350). The remaining abstracts were screened by two raters (AW, ChS) to determine their relevance to this review. A total of 12,992 studies were excluded as both raters deemed them irrelevant (1st step). The full texts of 214 articles were assessed for eligibility (2nd step) by five raters (AB, AW, ChS, CoS, DG), and seven studies describing universal prevention of NSSI were found (see PRISMA flow diagram, Figure 1). Due to (a) a wide range of outcome measures related to NSSI and significant heterogeneity in assessment methods, (b) the absence of controlled conditions, (c) the absence of power analyses to justify sample sizes, and generally low sample sizes to detect effect sizes typical for universal prevention, and (d) weak quality ratings in all studies according to the Effective Public Health Practice Project (EPHPP) risk of bias criteria (see Supplementary Methods), the number of studies was considered too small, heterogeneous and weak in quality to conduct a meaningful meta-analysis.

## Results

According to our systematic literature research, seven studies have established and performed universal prevention of NSSI with an adolescent target group. Within these studies, there was high heterogeneity in study and sample characteristics (see Table 1). While Baetens et al. (23) conducted a cluster-randomized controlled trial (class-wise, pre-post measurement), the other studies used a pre-post design with a maximum follow-up of 12 months. Three studies (24–26) assessed programs preventing NSSI and suicidal ideation, choosing a gatekeeper training approach. Gatekeeper programs train individuals (staff or students) who have regular face-to-face contact with a targeted group to recognize at-risk students early and direct them torwards professional help (27). Groschwitz et al. (25) aimed to advance NSSI prevention at schools via training of school staff in Germany (*n* = 257), and Robinson et al. (26) trained school social workers in Australia with similar intent (*n* = 213, mean age = 42.5 years). Cipriano et al. (24) selected eight 8th grade pupils as peer educators who conducted a peer education intervention for 6th and 7th grade pupils (*n* = 68, mean age = 11.59 years). Two studies aimed to change knowledge and attitudes about NSSI (i.e., “psychoeducation”) and to improve confidence and distress-coping when dealing with affected pupils (25, 26). Both studies reported positive effects on gatekeepers’ knowledge, attitudes, emotions, and awareness of problem-solving skills, yet did not evaluate whether these changes reduced NSSI behavior among adolescents in the particular school setting. The uncontrolled study by Cipriano et al. (24) reported significant changes in emotion regulation, self-esteem, body image, and maturity fears at post-treatment, after 5 weeks with 2-h sessions per week conducted online during school. In addition, there were no new cases of NSSI reported at the post-measurement. Shabbir et al. (28) surveyed the prevalence of NSSI and suicidal ideation in government and private schools in New Delhi and the impact of an education booklet on self-harm prevention for adolescents (*n* = 79, age 16–17 years). The authors reported an increased knowledge regarding non-suicidal self-harm from pre- to posttest but did not assess the occurrence of non-suicidal self-harm. A Master thesis by Byrum (29) performed a brief school-based prevention program [combining approaches of cognitive-behavioral therapy (CBT) with those of dialectical behavior therapy (DBT)], in order to reduce stress and dysfunctional behaviors in Mexican students (*n* = 79, mean age = 12.4 years). The program did not decrease negative stress-coping behaviors or perceived stress from pre- to posttest. NSSI was measured with one item (namely by asking the question “Do you cut yourself?”), but detailed information about frequency or type of NSSI was not reported, even when we specifically requested this information from the authors. Such information would have been vital, as the number of students engaging in “cutting” doubled (from 6 to 12) for the intervention group from pre- to posttest.

**TABLE 1:** Study characteristics of the studies included for the review.

References	Country	Target group	Study design	*N*	Mean age (SD) or age range, sex	Intervention	Duration of intervention	Main findings on NSSI
(23)	Belgium	Secondary school pupils	randomized pre-post	651	*M* = 12.85 (± 0.76), 49.8% female	In-classroom module: Happyles (in-classroom educational program focusing on general mental well-being and social connectedness) vs. HappylesPLUS module (with an additional 1-h psychoeducation on NSSI) Implementation: teacher	Two class periods of each 50 min	Pre-survey: 14.9% with a lifetime history of NSSI (girls > boys); mean onset of NSSI at 11.34 years (SD 2.14); cutting as most common NSSI method (44.1%). Post-intervention: no iatrogenic effects; no significant group difference regarding NSSI; incidence rate of 6%; among students with history of NSSI less perceived probability of future engagement in NSSI acts in both groups.
(29) (master thesis)	Mexico	Secondary school pupils	controlled pre-post	79	*M* = 12.40 (± 0.56), 54.4% female	Combination of preventive CBT and DBT techniques to reduce self-injuring behaviors, e.g., cutting Implementation: M.sc. psychology student	Three sessions over 3 days of each 90 min	Questionable iatrogenic effects: majority (18.99%) of the final sample indicated they had cut themselves to relieve stress; number of “self-cutting” adolescents doubled for the intervention group. Lacking information on how deliberate self-harm (DSH) was assessed.
(24)	Italy	Gatekeepers: peers (8th grade) and secondary school pupils	uncontrolled pre- and post	68	*M* = 11.59 (± 0.63) 38.2% female	Online education program with trained peer coaches, focused on improving emotion regulation abilities, enhancing feelings of self-esteem, and fostering a positive relationship with one’s own body image to reduce NSSI based on the psychoanalytic tradition Implementation: 8th grade pupils	Five sessions per week of each 120 min during school hours	Post-intervention: statistically significant improvement in emotion regulation abilities (acceptance of one’s emotions, access to effective emotion regulation strategies), higher self- esteem and lower scores on personal alienation, body dissatisfaction and fear maturity.
(25)	Germany	Gatekeepers: school staff of secondary schools	uncontrolled pre-post, 6 months follow-up	267	Not specified, 82.1% female	Workshop for school staff (teachers, social workers, and psychologists) to increase knowledge on NSSI and suicidality Implementation: school staff	2-day workshop of 7-h each	Post-intervention: large effect sizes for improvement in confidence and perceived knowledge (highest increase among teachers); significant decrease of negative attitudes toward NSSI; high satisfaction with the program (highest among teachers); differences between professions; largest difference in knowledge about the place where to seek help for adolescents with NSSI.
(30)	USA	Secondary school pupils	uncontrolled pre-post	282	*M* = 16.07 (± 1.32), 48.5% female	In-classroom module: psychoeducational “Signs of Self-Injury Program” (SOSI) to increase knowledge, improve help-seeking attitudes and behaviors, and decrease acts of NSSI Implementation: school staff	One class period of 50 min	Pre-survey: 25.9% with a lifetime history of NSSI; mean score for frequency 3.21 (SD = 1.89); over 10% had engaged in at > 1 act of NSSI in the month prior to the survey (range = 2.4–20.0%); 70.0% indicated someone knew about the self-injury, most often a friend (35.9%); 46.24% had > 1 friend who engages in NSSI. Post-intervention: no iatrogenic effect; increased accurate knowledge + improved help-seeking attitudes and intentions among students; no specific effects on NSSI acts measured.
(26)	Australia	Gatekeepers: school welfare staff/teachers of secondary schools	uncontrolled pre-post, 6 months follow-up	213	*M* = 42.5 (± 10.6), 85.9% female	Information on the epidemiology of DSH, the relationship to suicide and interventions. Inclusion of case vignettes Implementation not mentioned	1- or 2-day workshop of each 7 h	Post-intervention: significant positive effect on both perceived confidence, attitudes and skill when dealing with DSH; increase of knowledge especially for participants with low knowledge in the beginning.
(28)	India	Secondary school pupils	uncontrolled pre-post	79	“Majority 16 to 17 years”, 24.5% female	Information booklet on knowledge of strategies to prevent non-suicidal self-harm	One-time	Pre-intervention: marginally higher prevalence of reported incidents of NSSI in government schools, risk of self-harm significantly higher in private schools, impulsiveness only significant risk factor; no correlation between non-suicidal self-harm and demographic and socio-economic characteristics. Post-intervention: increase of knowledge on DSH; assessment of associated risk factors; higher level of knowledge on NSSI and its prevention, higher level among private school compared to government school pupils.

Only the further two universal prevention programs used valid questionnaires to measure the occurrence of NSSI in pupils. The “Signs of Self-Injury” (SOSI) psychoeducational program by Muehlenkamp et al. (30) attempts to increase knowledge, improve help-seeking behaviors, and decrease NSSI. An uncontrolled pre-post evaluation including five schools (*n* = 274; mean age = 16.07 years) implemented the program in selected classrooms (four schools with smaller classes with “at risk” pupils having emotional/behavioral problems, one school with “health” classes). Although SOSI significantly improved knowledge about NSSI, help-seeking attitudes and intentions, no significant changes were found in regards to self-reported formal help seeking actions.

Baetens et al. (23) examined differences between the programs Happyles and HappylesPLUS in 651 Belgian school pupils (mean age = 12.85 years) using a cluster-randomized (class-wise) pre-post design. Happyles is an in-classroom educational prevention program tailored to enhance general mental well-being and social connectedness and was implemented in the control group. HappylesPLUS additionally incorporated a 1-h psychoeducation module on NSSI, which was offered to participants of the intervention group. Both groups did not significantly differ in terms of the incidence of NSSI, and they both self-reported a reduced likelihood of potential future engagement in NSSI as well as an increased emotional awareness (23).

For an overview of the study characteristics of the seven studies included, see Table 1.

## Discussion

To the best of our knowledge, this is the first systematic review exploring universal prevention programs targeting NSSI. We only identified seven studies in total of which only two studies (23, 30) reported the incidence of NSSI behaviors (occurrence, frequency, and/or method) post-prevention. These studies observed no significant changes in actual NSSI behaviors. It is regrettable that, according to EPHPP criteria, all studies identified are characterized by a weak study quality rating due to several methodological limitations. Many of these limitations are discussed by the authors themselves: Muehlenkamp et al. (30) state that they had a rather small study sample, without a control group or evaluation of faculty or staff. Baetens et al. (23) refer to a lack of long-term follow-up data, insufficient blinding of conditions, and a significant diversity in school climate and stigmatization of psychological symptoms within the participating schools, without taking those factors into account for their analysis.

One study Byrum (29) reported increased numbers of NSSI post-prevention. A lack of information on the measurement, frequency or type of self-injury during and post-intervention hindered definitive conclusions regarding changes in NSSI behavior to be drawn. Such a lack of post measurements in most of the identified studies is concerning, as the primary goal of universal prevention is to reduce the incidence of mental disorders or high-risk behaviors (here: less NSSI). We suggest that it is essential to establish standards for prevention research, equivalent to good clinical practice (GCP) in clinical trials [e.g., (31)]. Given the high prevalence and the clinical impact of NSSI, the key result of our systematic review, namely a general scarcity of high-quality studies in the field of universal prevention of NSSI, is alarming. Below, we summarize crucial observations made during the literature review:

### Focus on selective/targeted prevention

For selective/targeted approaches, there is meta-analytical evidence that targeted prevention programs show moderate effects sizes in reducing NSSI [e.g., *d* = 0.51; *g* = −0.44 for DBT-A (17, 32)]. What are the reasons for the preponderance of studies in the field of targeted prevention compared to universal prevention of NSSI? The higher expected effect sizes for prevention in patients compared to universal prevention programs [where effect sizes have been recently shown to range between *d* = 0.07 and *d* = 0.40 (33)] may be one factor (34). Moreover, targeted prevention shows higher feasibility, as it can occur in a controlled, clinical setting. Thus, evaluation studies on universal prevention programs are more resource-intensive, requiring higher case numbers and often collaborations outside the clinic, such as with schools. However, from a salutogenetic perspective [Ottowa-Charta (35)], universal approaches are particularly important, as they strive to maintain health rather than reduce symptoms (34, 36). Furthermore, interventions at an early stage are considered cost-effective. Contrary to therapeutic interventions regarding NSSI, which are usually conducted in an in- and/or out-patient setting and entail high measurable expenses, preventive approaches may in case of success result in a reduction of clinical presentation and thus costs for the health system (37, 38).

Overall, research shows that universal programs are highly accepted among adolescents (39). One step to make universal programs more feasible and viable, despite their costs, could be to establish independent institutions solely dedicated to maintaining mental health (40).

### Focus on psychoeducation

To date, studies regarding NSSI prevention primarily work with psychoeducational elements to reduce the incidence of NSSI, however, so far without significant effects. Meta-analyses on universal prevention in anxiety, depression, or eating disorders showed that methods from cognitive behavioral therapy, skill training or cognitive dissonance seem to be more effective in reducing symptoms. By contrast, psychoeducational methods have even produced iatrogenic effects (e.g., in patients with eating disorders) (21, 41). Ultimately, a promising future avenue could be to strengthen protective factors that allow adolescents to improve their ability to manage their everyday lives. It might be worthwhile to focus on improving emotion regulation in order to strengthen protective factors and improve adolescents’ management of their everyday lives rather than on merely mitigating possible risk factors.

### Limitations

The review may be countered with the critique that only two online databases (PubMed and Google Scholar) were searched for articles, possibly restricting the pool of references found. It may have been beneficial to search further databases, even if cursory searches of other data bases revealed no additional studies of interest and recent research demonstrated no major supplementary finding when searching comparable terms with only two databases versus with three (32). Given that 94.58% of the 13,955 identified studies were excluded, it remains an open question whether the eligibility criteria and the related research focus (NSSI only) were too narrow. The initial decision to include self-harm as search term when setting focus specifically on NSSI may have contributed to the many results found.

The decision to focus on NSSI prevention was deliberately reached due to the deficiency of literature on this subject. Nevertheless, owing to the small number of suitable NSSI prevention studies found, the question is raised whether the review might have benefited from including a wider range of studies that represent the state of the art. A suggestion for future meta-analyses could be not to specify NSSI so narrowly, but to expand it to include self-harm and to encompass studies that concurrently focus on socio-emotional processes that are theoretically assumed to underlie NSSI/self-harm [e.g., emotion regulation (42, 43)]. In this context, potential impacts on NSSI/self-harm could also be examined. However, since the socio-cognitive and emotional mechanisms underlying NSSI/self-harm are largely undefined as yet, it is difficult to determine which prevention programs, with which socio-emotional focus, pertain to NSSI/self-harm and how strong these associations are.

## Conclusion

The concerning key result of our systematic review is not only the scarcity of studies evaluating universal approaches of NSSI prevention, but also their low quality in terms of measurement, risk of bias, and reporting of results. It’s alarming that none of the studies on this topic demonstrated effectiveness in preventing NSSI. Consequently, there is currently no evidence base for effective universal prevention of NSSI in youth, even though the prevalence of NSSI among adolescents is high and the importance of this field of research is increasingly emphasized in contemporary research (4, 6, 30, 44).

In conclusion, initial research is promising and suggests that the approach to tackle NSSI via universal prevention is meaningful. Yet, high-quality studies on the development and evaluation of universal NSSI prevention in adolescents are urgently needed, and in ongoing work, we aim to contribute to closing this gap in the literature (45).

## Author contributions

AB conceived the idea for the systematic review. ChS, AW, DG, and CvS performed the literature search. AB and ChS removed duplicates and screened for eligibility. AB and AR interpreted the results. AB, ChS, CvS, and AR wrote the manuscript. AS, SK-S, and MR critically revised the manuscript. All authors read and approved the final manuscript.
