# Supplementary material for: Universal prevention for non-suicidal self-injury in adolescents is scarce - A systematic review
Source: Front Psychiatry. 2023 Oct 23;14:1130610. doi: 10.3389/fpsyt.2023.1130610 (PMC10627158; doi:10.3389/fpsyt.2023.1130610)
Supplement: Supplementary file 1 [file Table_1.pdf]

Supplementary Materials

Search string PubMed:

(("non-suicidal self-injury" [All Fields] OR "self-injury" [All Fields] OR "self-harm" [All Fields] OR “NSSI” [All Fields] OR ("suicide"[All Fields])) AND (prevent* [All Fields]) AND (adolescen* [All Fields] OR child* [All Fields] OR youth [All Fields]). AND ("2023/03/23"))

Indexing Google Scholar:

“non-suicidal self-injury”, “self-injury”, “self-harm”, “NSSI”, “suicide”, “prevention”, “adolescent”, “adolescence”, “child”, “children“, “youth”
[Date - Publication]: 1960 - "2023/03/23"

Quality Assessment

The overall study quality was evaluated according to the Canadian Effective Public Health Practice Project (EPHPP) recommendations on the domains selection bias, study design, confounders, blinding, data collection methods, withdrawals and dropouts (Project 1998). The global study quality was determined in the next step depending on ratings of the particular components mentioned above and then defined as weak, moderate, or strong. Studies without areas rated as weak were deemed as “strong”. One weak area led to a rating of “moderate” quality. Studies with two or more weak domains were classified as “weak”.

Study Quality

The assessment of respective study qualities via the Canadian Effective Public Health Pratice Project (EPHPP) quality assessment tool (Evans, Lasen et al. 2015) showed an overall weak study quality for all seven studies with distinct deficiencies in ratings of study design, confounders, and blinding. Considering that all studies used a pre-post-design and already

received a weak rating for this category, it needed only one further weak or moderate rating in the composition of the study to be rated as overall weak. Most of the studies were rated as weak for four components (Robinson, Gook et al. 2008, Muehlenkamp, Walsh et al. 2010, Groschwitz, Munz et al. 2017, Cipriano, Apera et al. 2022), two studies in five domains (Byrum 2019, Shabbir, Kapoor et al. 2021); Baeten's pilot study was rated as strong in three and as weak in two categories (Baetens, Decruy et al. 2020). Regarding the cumulative evidence, there is a high risk of publication bias considering that uncontrolled studies are easy to conduct. Additionally, there may be selective reporting within studies as to whether samples from the same group were truly independent. Studies also possibly lacked reporting adherences to protocols and/or blinding of raters.

For individual study quality ratings according to the EPHPP, see Table 1.

Authors	Selection bias	Study design	Confounders	Blinding	Data methods	Withdrawals/dropout	Total
Baetens et al., 2020	Strong	Weak	Weak	Moderate	Strong	Strong	**Weak**
Byrum, 2019	Moderate	Weak	Weak	Weak	Weak	Moderate	**Weak**
Cipriano et al., 2022	Weak	Weak	Weak	Weak	Moderate	Moderate	**Weak**
Groschwitz et. al., 2017	Moderate	Weak	Weak	Weak	Moderate	Strong	**Weak**
Muehlenkamp et al., 2010	Moderate	Weak	Weak	Weak	Strong	Strong	**Weak**
Robinson et al., 2008	Moderate	Weak	Weak	Weak	Moderate	Moderate	**Weak**
Shabbir, Kapoor & Biswas, 2021	Strong	Weak	Weak	Moderate	Weak	Weak	**Weak**

Table 1: Study quality ratings according to the EPHPP
